# CSF IL-8 Associated with Response to Gene Therapy in a Case Series of Spinal Muscular Atrophy

**DOI:** 10.1007/s13311-022-01305-9

**Published:** 2022-10-26

**Authors:** Sumit Verma, Kelsey Perry, Raj Razdan, J. Christina Howell, Alice L. Dawson, William T. Hu

**Affiliations:** 1 Department of Pediatrics, Emory University School of Medicine, Children’s Healthcare of Atlanta, Atlanta, GA 30324 USA; 2 Department of Neurology, Emory University School of Medicine, Children’s Healthcare of Atlanta, Atlanta, GA 30324 USA; 3 Department of Neurosciences, Children’s Healthcare of Atlanta, Atlanta, GA 30324 USA; 4 Health Care Policy, and Aging Research, Rutgers-Robert Wood Johnson Medical School and Rutgers Institute for Health, New Brunswick, NJ 08901 USA; 5 Rutgers-Robert Wood Johnson Medical School, 125 Paterson Street, Suite 6200, New Brunswick, NJ 08901 USA

**Keywords:** Nusinersen, Weight, CHOP-INTEND, Motor CMAP, Neurofilament light chain, Interleukin-8

## Abstract

Gene therapies have greatly changed the outlook in spinal muscular atrophy (SMA), and this disorder provides a rare opportunity to study longitudinal biomarker changes correlated with reduced disease burden and improved clinical outcomes. Recent work suggests clinical response to correlate with declining cerebrospinal fluid (CSF) levels of the neurodegenerative marker neurofilament light chain (NfL) in children receiving serial anti-sense oligonucleotide therapy. However, change in CSF NfL levels is no longer a practical biomarker as more children undergo single-dose gene replacement therapy. Here we leverage serial CSF samples (median of 4 per child) collected in 13 children with SMA undergoing anti-sense oligonucleotide therapy to characterize the longitudinal profiles of NfL as well as inflammatory and neuronal proteins. In contrast to neurodegeneration in adults, we found NfL levels to first decrease following initiation of treatment but then increase upon further treatment and improved motor functions. We then examined additional CSF inflammatory and neuronal markers for linear association with motor function during SMA treatment. We identified longitudinal IL-8 levels to inversely correlate with motor functions determined by clinical examination (*F*(1, 47) = 12.903, *p* = 0.001) or electromyography in the abductor pollicis brevis muscle (*p* = 0.064). In keeping with this, lower baseline IL-8 levels were associated with better longitudinal outcomes, even though this difference diminished over 2 years in the younger group. We thus propose CSF IL-8 as a biomarker for baseline function and short-term treatment response in SMA, and a candidate biomarker for future treatment trials in other neurodegenerative disorders.

**Supplementary Information:**

The online version contains supplementary material available at 10.1007/s13311-022-01305-9.

## Introduction

Spinal muscular atrophy (SMA) is a congenital neurodegenerative disorder which results in progressive loss of motor function and death. SMA is caused by mutations in the survival motor neuron (*SMN1*) gene which result in reduced SMN protein levels [1], and has been heralded as a success in molecular medicine with the introduction of gene modulating or replacement therapy. The anti-sense oligonucleotide nusinersen targets the exon splicing of a related *SMN2* gene to elevate levels of functional SMN protein [2], and is serially administered through intrathecal injections. On the other hand, *SMN1*-expressing adeno-associated virus 9 vector onasemnogene abeparvovec-xioi directly enhances SMN protein levels and is administered as a one-time intravenous administration [3]. Children treated with either therapy showed a range of motor outcomes, and it remains incompletely understood which children would respond favorably to either or both therapies. Fluid biomarkers associated with longer-term response to treatment, especially if linked to neuropathologic burden, may improve our understanding of variability in response to treatment, and inform the choice of repeated or one-time therapy. Increased uptake of the one-time intravenous therapy, however, means fewer opportunities to assess brain biomarker profiles in SMA children.

SMN levels would be the ideal biomarker for target engagement, but no soluble form of the protein is reliably detected in the cerebrospinal fluid (CSF) or blood [3]. There is also a normal development-related reduction of spinal cord SMN* in* *utero* and during the first 3 months of life [4]. These findings diminish the prospect of developing SMN as a viable biomarker. Because nusinersen therapy requires serial intrathecal injections, its clinical use provides a unique opportunity to analyze serial CSF samples in children with SMA for biomarker discovery. Previously, treatment-associated temporal decline in CSF neurofilament light chain (NfL) levels *at the group level* was associated with improved motor function assessed by the Children’s Hospital of Philadelphia Infant Test of Neuromuscular Disorders (CHOP INTEND) score [5]. This led to the proposal of CSF NfL as a marker of treatment response [6, 7]. However, older children in that series showed attenuated improvement despite similar reduction in NfL levels, suggesting that NfL reduction may only confer prognostic information in younger children or represent an epiphenomenon of treatment.

Other CSF proteins — including those implicated in inflammation and neuronal activity — have also been proposed as candidate disease markers in adult spinal neurodegenerative disorders such as amyotrophic lateral sclerosis (ALS). These have not been systemically examined in SMA, and we hypothesized that CSF proteins involved in inflammation and neuronal activity may better correlate with individual response to *SMN1* enhancing therapies than NfL [8–10]. Here we collected and analyzed 49 CSF samples from nine younger (≤ 24 months of age; median 12 months) and four older (> 24 months of age; median 137 months) children receiving nusinersen to identify proteins temporally associated with clinical outcomes as well as proteins whose baseline levels complement other measures in predicting response to therapy.

## Materials and Methods

The study was conducted in accordance to the ethical standards and policies of Emory University, Children’s Healthcare of Atlanta, and US 45 CFR Part 46, and with the Helsinki Declaration of 1975 (as revised in 2000). It was approved by the Children’s Healthcare of Atlanta Institutional Review Board (#13-151), and written informed consents were obtained from the parents or legal guardians of all children. Ten newly diagnosed (nine younger and one older) and three older previously diagnosed study participants due to start on nusinersen therapy were consecutively recruited between March 2017 and January 2020 (Table 1). One younger child died due to respiratory failure during the study period, and autopsy was declined by the parents. One older child had a precipitous decline before improving (CHOP INTEND score drop from 47 to 19 requiring prolonged hospitalization, eventually recovering to a score of 28), and excluding this child from subsequent analyses gave similar findings except the baseline CHOP INTEND was higher in the younger group (*p* < 0.001). Because all three other older children and two younger children also experienced significant decline in CHOP INTEND scores (≥ 30% from previous visit; three transiently and two permanently), we included all children in this study.

**Table 1:** Baseline characteristics of the study participants, with median values and range shown for continuous variables and group-based differences evaluated by Fisher’s exact test or Mann–Whitney *U* tests

**Characteristics**	**Younger cohort**	**Older cohort**	***p***
*N*	9	4	
Female	5 (56%)	2 (50%)	1.000
Age at onset (months)	2.8 (1.1, 6.4)	5.3 (1.6, 17.2)	0.414
Feeding tube	4 (44%)	4 (100%)	0.105
BiPAP use	4 (44%)	4 (100%)	0.105
Treatment baseline			
Age (months)	12.1 (6.3, 21.0)	137 (44, 213)	0.003
Weight (kg)	6.70 (3.6, 8.0)	15.5 (11.1, 28.3)	0.003
CHOP INTEND scores	25 (14, 40)	11.5 (8, 47)	0.199
CMAP_APB_ (mV)	0.70 (0.30, 1.30)	0.95 (0.10, 1.80)	1.000
CMAP_ADM_ (mV)	0.30 (0.20, 0.80)	0.45 (0.10, 1.12)	0.940
CSF biomarkers			
NfL (ng/mL)	7.16 (3.37, 18.20)	2.10 (2.04, 3.18)	0.024
Fractalkine (pg/mL)	34.3 (11.2, 64.3)	38.0 (33.0, 55.0)	0.381
IL-8 (pg/mL)	17.3 (10.4, 43.6)	18.6 (16.8, 23.9)	0.548
IP-10 (pg/mL)	217 (145, 468)	203 (124, 292)	0.714
MCP-1 (pg/mL)	751 (445, 1007)	666 (460, 705)	0.714
sAPPα (pg/mL)	108 (92, 215)	149 (98, 166)	0.714
sAPPβ (pg/mL)	178 (111, 307)	233 (1, 267)	0.905

Clinical measures including age (in months), weight (in kg), and CHOP INTEND scores were serially collected. Compound muscle action potential (CMAP) from two right hand muscles (abductor digiti minimi or ADM, and abductor pollicis brevis or APB) was also obtained during electromyography performed by a board-certified pediatric neuromuscular specialist (SV; [11, 12]). Before each intrathecal nusinersen infusion, 5 mL of CSF was collected into sterile polypropylene tubes, labeled, frozen, and stored at −80 °C within 60 min. CSF samples were then batch-transferred for analysis.

For biomarkers, CSF samples were analyzed using an enzyme-linked immunosorbent assay (ELISA, 10-7002, Uman Diagnostics, Umeå, Sweden) for NfL. Four inflammatory biomarkers were analyzed in a Luminex 200 platform (Milliplex MAP HCYTOMAG-60 K, MilliporeSigma, Burlington, MA) based on prior associations with SMA or related motor neuron disease. These include monocyte chemoattractant protein 1 (MCP-1) linked to multiple cell types but specifically astrocyte in SMA [10]; fractalkine/CXCL3 related to microglial toxicity including in motor neuron disease and spinal cord injury [13–15]; interferon gamma-induced protein 10 or IP-10/CXCL-10 associated with T_H_1 inflammatory response [16, 17]; and interleukin-8 (IL-8/CXCL-8) implicated in T_H_17 inflammatory pathways [18–22]. Soluble forms of the amyloid precursor protein sAPPα and sAPPβ were also measured in the Luminex 200 platform (Immuno-Biological Laboratories, Minneapolis, MN) due to their implication in ALS [23, 24]. All assays were performed using manufacturers’ protocols with the exception that 100 mL of CSF was used in the inflammatory protein assays, and these assays have been used extensively in the Hu Lab to characterize neurodegenerative, inflammatory, and other biological processes in aging, Alzheimer’s disease (AD), multiple sclerosis (MS), HIV-associated neurocognitive dysfunctions, and COVID-19-related neurological dysfunctions with high accuracy and precision [22, 25–28].

Statistical analyses were conducted using SPSS Version 26.0 (Armonk, NY). Descriptive statistics for each variable were reported. Fisher’s exact tests (for categorical variables) or Mann–Whitney *U* tests (for continuous variables) were used to identify differences between the two subgroups. CSF biomarker levels were first log_10_ transformed due to non-normal distribution, and then Z-transformed using the whole group’s distribution for comparison between different biomarkers’ variances.

Analysis of long-term clinical outcome was performed using linear mixed modeling to account for intra-subject correlation between adjacent timepoints. The main clinical outcome (CHOP INTEND score) was entered as the dependent variable; age category (≤ 24 months, > 24 months), sex, corresponding baseline motor measures, and age category × time were entered as fixed variables; and time was also entered as a random variable, with *p* < 0.01 to adjust for multiple comparisons. To discover if serial CSF analyte levels temporally correlated with CHOP INTEND, time-varying CSF analyte levels and their interaction terms with time were also added as fixed variables. False discovery rate of 5% was used to adjust for multiple hypothesis testing across the seven analytes using the Benjamini–Hochberg method. For each CSF analyte, the lesser *p*-value from the analyte or analyte × time term was compared against the FDR-related thresholds.

Finally, to test if baseline levels of four analytes longitudinally correlated with motor outcomes associated with subsequent motor outcomes in the younger cohort, sex, sex × time, baseline analyte, and baseline analyte × time were entered as fixed variables, and time was entered as fixed as well as random variables. Akaike information criterion (AIC) was used to select between different models.

## Results

**Fig. 1:**
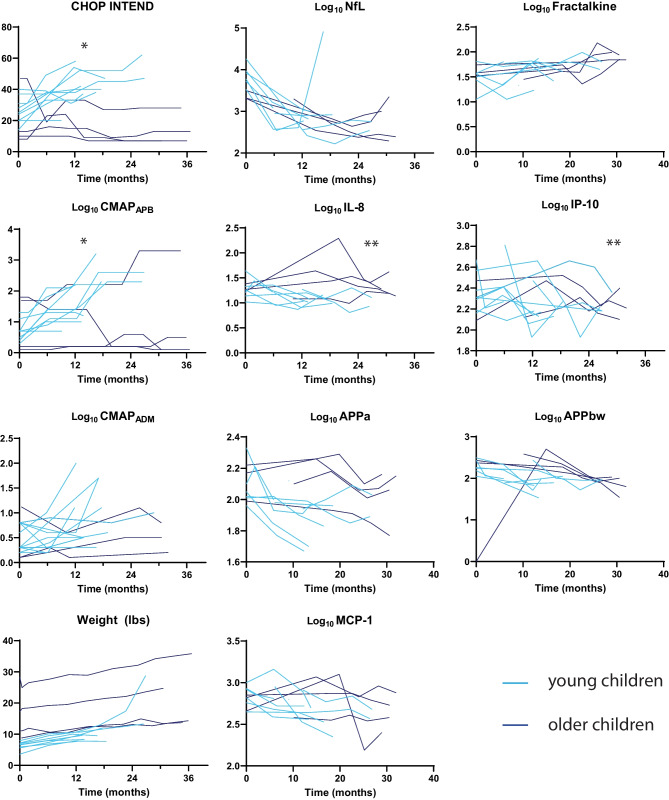
Longitudinal trends of clinical and fluid biomarkers in SMA children treated with nusinersen. Compared to older children (*n* = 4, dark blue), younger children (*n* = 9, light blue) had greater CHOP INTEND and CMAP_APB_ over time (*p < 0.005), but not CMAP_ADM_ and weight. In the CSF, levels of NfL, MCP-1, sAPPα, and sAPPβ declined in levels regardless of age, but levels of IL-8 and IP-10 only decreased in younger children (**p < 0.05)

Compared to the older group, younger children with SMA had lower baseline weight (*p* < 0.003), higher baseline NfL levels (log_10_-transformed for non-normal distribution at baseline, *p* = 0.024), and better motor outcomes by CHOP INTEND or EMG (Fig. 1; Supplementary Table 1). All 9 children starting treatment under the age of 24 months had significant improvement (but one sudden death), while all children starting treatment after age 11 had poor response. One child starting treatment at 44 months of age had improvement in EMG similar to the younger children, but this child’s CHOP INTEND scores never exceeded 30. Because of divergent treatment outcomes between younger and older children, age grouping was introduced as a variable in all subsequent analyses.

With nusinersen treatment, CSF NfL levels declined in younger and older children, but the initial decline in NfL levels was followed by an increase in eight patients (five younger and three older; median 19 months after treatment initiation, range 5.8–24 months). The longitudinal trajectory of NfL was best modeled by the equation log_10_(NfL) = log_10_(NfL_T=0_) − 0.116 × log10(NfL_T=0_) × Time + 0.010 × Time^2^, with the initial rate of decline proportional to baseline NfL levels (e.g., higher baseline NfL levels had greater rates of decline) but the subsequent increase independent of baseline NfL levels or age group.

**Fig. 2:**
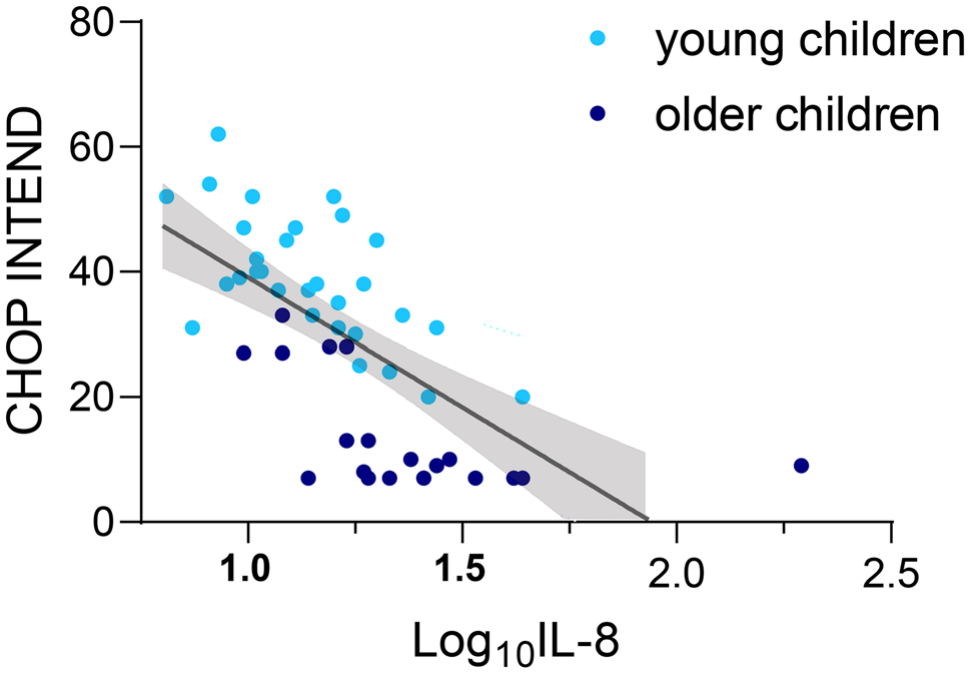
Relationship between CSF IL-8 levels and CHOP INTEND scores. Values from all times in younger (light blue) and older (dark blue) children were plotted, and a single regression line better fits all data than one line for each age group

Because higher baseline CSF NfL levels — hypothesized to reflect neurodegeneration — paradoxically associated with better baseline CHOP INTEND scores and its later levels rose with age, we sought to identify other CSF biomarkers correlated with disease severity and treatment response. Levels of MCP-1 (0.035/mo, 95% CI 0.004–0.066, *p* = 0.30), sAPPα (0.055/mo, 95% CI 0.016–0.099, *p* = 0.012), and sAPPβ (0.054/mo, 95% CI 0.034–0.075, *p* < 0.001) all declined over time independent of age and thus treatment response. In contrast, levels of IL-8 (0.089/mo, 95% CI 0.008–0.171, *p* = 0.031) and IP-10 (0.068/mo, 95% CI 0.010–0.125, *p* = 0.023) declined only in the younger group with better treatment response. Fractalkine was the only analyte which increased over time (0.046/mo, 0.017–0.075, *p* = 0.004). Examining time-dependent relationships between CSF analyte levels and clinical outcome measures (Supplementary Table 2) showed IL-8 (*p* < 0.001, Fig. 2), MCP-1 (*p* = 0.012), and sAPPα (*p* = 0.028) to longitudinally associate with CHOP INTEND scores independent of age grouping. IP-10 (*p* = 0.008) and IL-8 (*p* = 0.064) also correlated with longitudinal CMAP in the ADM and APB. Thus, the longitudinal linkage between IL-8 and independent clinical measures (global function and regional CMAP) supports this CSF protein as a candidate biomarker for SMA disease severity.

**Fig. 3:**
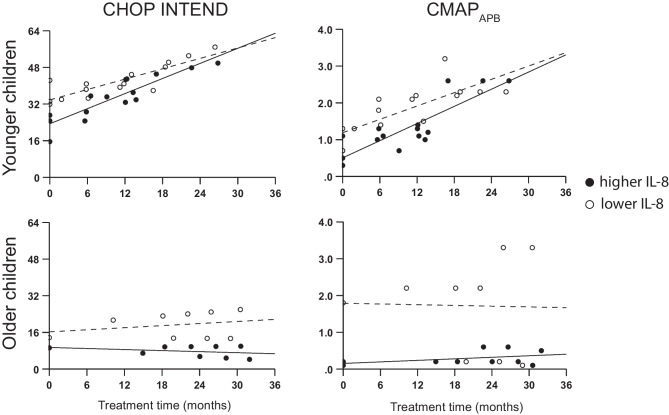
Predicted CHOP INTEND and CMAP_APB_ based on fixed effects according to age and baseline IL-8. Lower baseline IL-8 levels (< 18.40 pg/mL, open circles and dashed line) were consistently associated with better outcomes than higher baseline IL-8 levels (filled circles and solid line)

Even though IL-8 levels correlated with clinical outcomes over time, the need for serial CSF collection reduces its practical value. We thus examined if baseline demographic variables and biomarker levels (IL-8 and NfL) were associated with longitudinal clinical outcomes. Younger age at treatment initiation was associated with greater improvement in CHOP INTEND (*p* < 0.001), CMAP_APB_ (*p* < 0.001), and age- and sex-adjusted weight Z-score (*p* < 0.001, Table 2). Lower baseline IL-8 levels were additionally associated with better CHOP INTEND scores and CMAP_APB_ regardless of treatment initiation age, but the difference diminished over time for younger children compared to older children (Fig. 3). On the other hand, lower baseline NfL was associated with worse weight at baseline for both age groups, but associated with greater weight gain in the younger group only.

**Table 2:** Effects of baseline factors on subsequent clinical outcomes. Interaction terms are listed in order of *F* values for main effect, with interaction terms having the greatest *F* value listed first

	***B* (95% CI)**	***p***
**Outcome: CHOP INTEND**		
Time (months)	0.090 (−0.189, 0.368)	0.521
Younger age	10.76 (−0.60, 22.11)	0.061
Female	−0.338 (−7.194, 6.518)	0.917
Baseline CHOP INTEND	0.536 (−0.116, 1.188)	0.095
Baseline Zlog_10_(IL-8)	−5.396 (−13.662, 2.870)	0.176
Baseline Zlog_10_(NfL)	−3.726 (−8.265, 0.812)	0.097
Younger age × Zlog_10_(IL-8) × Time	0.767 (0.407, 1.126)	< 0.001
Older age × Zlog_10_(IL-8) × Time	−0.421 (−0.939, 0.095)	0.107
Female × Younger age × Time	1.088 (0.689, 1.488)	< 0.001
Male × Younger age × Time	0.281 (−0.133, 0.696)	0.178
Female × Older age × Time	−0.108 (−0.537, 0.320)	0.614﻿
Male × Older age × Time	Reference	
**Outcome: CMAP**_**APB**_		
Time (months)	0.049 (0.016, 0.083)	0.005
Younger age	0.235 (−0.081, 0.551)	0.140
Baseline CMAP_APB_	0.887 (0.589, 1.186)	< 0.001
Baseline Zlog_10_(IL-8)	0.005 (−0.242, 0.251)	0.969
Baseline Zlog_10_(IL-8) × Time	−0.067 (−0.097, −0.036)	< 0.001
Baseline CMAP_APB_ × Time	−0.117 (−0.182, −0.052)	< 0.001
Female × Younger age × Time	0.078 (0.033, 0.124)	0.001
Male × Younger age × Time	0.109 (0.064, 0.154)	< 0.001
Female × Older age × Time	0.134 (0.032, 0.235)	0.011
Male × Older age × Time	Reference	
**Outcome: weight Z-score**		
Time (months)	0.111 (0.062, 0.159)	< 0.001
Younger age	−1.275 (−2.454, −0.096)	0.035
Baseline Weight Z-score	1.180 (1.030, 1.330)	< 0.001
Baseline Zlog_10_(NfL)	0.558 (−0.343, 1.460)	0.220
Younger age × Time	0.114 (0.048, 0.180)	﻿< 0.001
Older age × Time	Reference	
Zlog_10_(NfL) × Time	−0.076 (−0.126, −0.027)	0.003

## Discussion

Gene modulating and replacement therapies have revolutionized the treatment of SMA. If most newly diagnosed SMA patients’ parents opt for one-time gene replacement therapy, only clinical measures and blood NfL levels would be available to forecast variability in outcomes including poor improvement seen in older children. Here we leveraged serially collected CSF samples from children with a wide range of baseline functions and clinical outcomes to show that NfL levels were paradoxically higher in children with better baseline functions and did not closely mirror clinical outcomes over time. We further identified IL-8 as a marker which tracked clinical response to treatment over time, and even its baseline levels were associated with short-term outcomes of younger and older patients. These findings suggest CSF IL-8 as a more meaningful biomarker related to or downstream of neurodegeneration than NfL in SMA and potentially other neurological disorders.

Consistent with a recent report in 68 children, we found younger age at treatment initiation to associate with better treatment responses [29]. In contrast to the prior biomarker study, however, we did not find NfL decline to confer outcome-specific information as NfL levels declined in both younger and older children despite the latter’s poor treatment response. CSF NfL has been proposed as a marker for general neurodegeneration in multiple sclerosis, Alzheimer’s disease, and other neurological disorders [30]. It was first modestly correlated with disease progression in MS [31], and its popularity in neurodegenerative diseases is fueled in part by a simple ELISA with readily detectable levels. Despite its promise as a marker for neuronal injury, CSF NfL levels only modestly differed between older adults with AD and normal cognition and serial sampling showed its levels to decrease — vs. increase like Tau or Aβ40 — over time in older adults [32, 33]. NfL’s time-dependent decrease in aging and Alzheimer’s disease is consistent with our observation of its development-associated increase and in support of NfL as a marker of neuronal integrity rather than neurodegeneration. We are aware of only one study from the Northeast ALS Consortium (NEALS) to have examined serial CSF NfL levels in ALS which found no appreciable annual change despite markedly elevated baseline levels [34]. This should not be surprising due to effects on CSF protein levels from cellular vulnerability to disease, proximity to perivascular space, and bulk flow dynamics beyond neurodegeneration. We thus add to prior caution that cross-sectional CSF NfL levels should be interpreted with care.

A number of CSF proteins analyzed here have each been previously suggested as biomarkers for ALS severity and progression [35–41]. In the same NEALS study which serially measured NfL levels in ALS [34], only two (MCP-1 and IL-18) out of sixty-five CSF cytokines evaluated were associated with rates of disease progression. MCP-1 was found in univariate analyses to associate with clinical outcomes in our study, but its effect gave way to IL-8 and basic demographic variables in multivariate analyses. IL-8 did not distinguish between ALS and non-ALS controls in the NEALS study, but two studies have found lower IL-8 to associate with better function in ALS [35, 36]. Aside from technical challenges in reliably measuring CSF cytokines, this discrepancy can be potentially explained by the fact that ALS and SMA are fundamentally different diseases. Whereas both are thought to represent motor neuron disorders, neuronal suppression of mutant ALS-causing gene products led to better function and survival than astrocytic suppression in animal models [42]. In contrast, greater functional recovery was noted in animal models of SMA when SMN is restored in astrocytes [43] than in motor neuron [44, 45]. It is thus possible that the differential impact of these two disorders on neurons and glia contribute to the distinct relationships between CSF IL-8 levels and clinical outcomes.

IL-8 is best known as a potent chemoattractant of neutrophils and is expressed at low levels by astrocytes, microglia, and neurons in disease-free brains [46]. IL-8 acts via its receptors CXCR1 and CXCR2, with the former highly expressed by primary human microglia and astrocytes while the latter by neurons in the brain and spinal cord (including elevated neuronal expression in ALS [47, 48]. CSF IL-8 levels are acutely increased in bacterial meningitis [20], traumatic brain/spinal cord injury [49, 50], and more recently neurological complications of COVID-19 [28, 51]. Among chronic disorders, CSF IL-8 levels are elevated in Alzheimer’s disease [25], multiple sclerosis [25, 52], and neuromyelitis optica [52], but reduced in HIV-associated neurocognitive dysfunction [22]. Even though astrocytes and microglia represent potential cellular sources underlying these changes [20, 53], lower IL-8 levels may variably represent decreased production (e.g., astrocytes returning to a basal state), increased uptake via CXCR1/2, or another pathologic process. For example, SMN depletion in mice resulted in retained introns and skipped exons, including genes of the IL-8 pathway [54]. In familial ALS associated with *C9orf72* hexanucleotide repeat expansion, stem cell-derived neurons demonstrate dysregulated cell cycle re-entry accompanied by increased IL-8 mRNA and protein levels [55]. In trauma, CSF IL-8 levels are influenced by blood brain barrier permeability [56]. In keeping with a potentially complex origin of IL-8 alteration, astrocytes also secrete MCP-1 (when non-stimulated) and IP-10 (when stimulated) whose levels did not correlate well with prognosis. At the same time, because a measured CSF biomarker level is influenced by multiple temporal and spatial processes, it is not straightforward to explain why one astrocytic protein better correlates with a clinical phenotype than another astrocytic protein. Nevertheless, the prognostic value of IL-8 reported here reinforces the need for further imaging- or single cell/nuclei-based analysis of astrocytic and microglial function in SMA, especially for the older children who showed persistently elevated IL-8 and poor clinical response to gene therapy.

Even though the number of children included here is comparable with previous longitudinal SMA biomarker studies [5], our sample size in this relatively rare disorder was still limited to permit broad generalization. CSF IL-8 level — at least at time of treatment initiation if not longitudinally — will therefore need to be measured in larger numbers of SMA children to validate it as a prognostic biomarker. There was also no biopsy-based or post-mortem neuropathologic analysis to determine the cellular (e.g., astrocytic vs. non-astrocytic) origin of CSF IL-8, making it difficult to speculate whether modulating IL-8 levels alongside *SMN* gene therapy would be beneficial. With these caveats in mind, CSF IL-8 and functionally related proteins should be further interrogated in children on intrathecal nusinersen for their clinical and mechanistic roles, and baseline CSF levels should be considered in children undergoing one-time intravenous gene replacement therapies.

## Conclusions

In children with SMA undergoing gene modulating therapy, CSF IL-8 levels longitudinally tracked response to therapy according to clinical assessment and electromyographic measures. In contrast, CSF NfL levels go through a decay followed by an increase independent of clinical response. Baseline CSF IL-8 levels should therefore be prospectively tested — among other fluid and physiologic markers — for stratifying children into better and worse responders when specialty services (e.g., pediatric EMG) are not available, as well as a pharmacodynamic biomarker in treatment trials of other neurodegenerative disorders including ALS.

## Supplementary Information

Below is the link to the electronic supplementary material.

Supplementary file1 (DOCX 18 KB)

Supplementary file2 (PDF 509 KB)

Supplementary file3 (PDF 535 KB)

Supplementary file4 (PDF 526 KB)

Supplementary file5 (PDF 544 KB)

Supplementary file6 (PDF 553 KB)

Supplementary file7 (PDF 518 KB)

## Abbreviations

AD: Alzheimer’s disease
ADP: Abductor digiti minimi
AIC: Akaike information criterion
ALS: Amyotrophic lateral sclerosis
APB: Abductor pollicis brevis
CHOP INTEND: The Children’s Hospital of Philadelphia Infant Test of Neuromuscular Disorders
CMAP: Compound muscle action potential
CSF: Cerebrospinal fluid
EMG: Electromyography
IL-8: Interleukin-8
IP-10: Interferon response protein 10
MCP-1: Monocyte chemoattractant protein 1
MS: Multiple sclerosis
NfL: Neurofilament light chain
sAPPα and β: Soluble amyloid precursor protein α and β
SMA: Spinal muscular atrophy

## References

[1] Kolb SJ, Coffey CS, Yankey JW, Krosschell K, Arnold WD, Rutkove SB (2017). Natural history of infantile-onset spinal muscular atrophy. Ann Neurol.

[2] Finkel RS, Mercuri E, Darras BT, Connolly AM, Kuntz NL, Kirschner J (2017). Nusinersen versus sham control in infantile-onset spinal muscular atrophy. N Engl J Med.

[3] Zaworski P, von Herrmann KM, Taylor S, Sunshine SS, McCarthy K, Risher N (2016). SMN protein can be reliably measured in whole blood with an electrochemiluminescence (ECL) immunoassay: implications for clinical trials. PLoS One.

[4] Ramos DM, d'Ydewalle C, Gabbeta V, Dakka A, Klein SK, Norris DA (2019). Age-dependent SMN expression in disease-relevant tissue and implications for SMA treatment. J Clin Invest.

[5] Olsson B, Alberg L, Cullen NC, Michael E, Wahlgren L, Kroksmark AK (2019). NFL is a marker of treatment response in children with SMA treated with nusinersen. J Neurol.

[6] Wurster CD, Steinacker P, Gunther R, Koch JC, Lingor P, Uzelac Z (2020). Neurofilament light chain in serum of adolescent and adult SMA patients under treatment with nusinersen. J Neurol.

[7] Faravelli I, Meneri M, Saccomanno D, Velardo D, Abati E, Gagliardi D (2020). Nusinersen treatment and cerebrospinal fluid neurofilaments: an explorative study on Spinal Muscular Atrophy type 3 patients. J Cell Mol Med.

[8] Sajjad MU, Blennow K, Knapskog AB, Idland AV, Chaudhry FA, Wyller TB (2020). Cerebrospinal fluid levels of interleukin-8 in delirium, dementia, and cognitively healthy patients. J Alzheimers Dis.

[9] Hu WT, Chen-Plotkin A, Arnold SE, Grossman M, Clark CM, Shaw LM (2010). Biomarker discovery for Alzheimer's disease, frontotemporal lobar degeneration, and Parkinson's disease. Acta Neuropathol.

[10] Martin JE, Nguyen TT, Grunseich C, Nofziger JH, Lee PR, Fields D (2017). Decreased motor neuron support by SMA astrocytes due to diminished MCP1 secretion. J Neurosci.

[11] Glanzman AM, McDermott MP, Montes J, Martens WB, Flickinger J, Riley S (2011). Validation of the Children's Hospital of Philadelphia Infant Test of Neuromuscular Disorders (CHOP INTEND). Pediatr Phys Ther.

[12] Verma S, Forte J, Ritchey M, Shah D (2020). Motor unit number index in children with later-onset spinal muscular atrophy. Muscle Nerve.

[13] Biber K, Neumann H, Inoue K, Boddeke HW (2007). Neuronal 'On' and 'Off' signals control microglia. Trends Neurosci.

[14] Cardona AE, Pioro EP, Sasse ME, Kostenko V, Cardona SM, Dijkstra IM (2006). Control of microglial neurotoxicity by the fractalkine receptor. Nat Neurosci.

[15] Freria CM, Hall JC, Wei P, Guan Z, McTigue DM, Popovich PG (2017). Deletion of the fractalkine receptor, CX3CR1, improves endogenous repair, axon sprouting, and synaptogenesis after spinal cord injury in mice. J Neurosci.

[16] Klein RS (2004). Regulation of neuroinflammation: the role of CXCL10 in lymphocyte infiltration during autoimmune encephalomyelitis. J Cell Biochem.

[17] Liu MT, Chen BP, Oertel P, Buchmeier MJ, Armstrong D, Hamilton TA (2000). The T cell chemoattractant IFN-inducible protein 10 is essential in host defense against viral-induced neurologic disease. J Immunol.

[18] Jin M, Günther R, Akgün K, Hermann A, Ziemssen T (2020). Peripheral proinflammatory Th1/Th17 immune cell shift is linked to disease severity in amyotrophic lateral sclerosis. Sci Rep.

[19] Aloisi F, Carè A, Borsellino G, Gallo P, Rosa S, Bassani A (1992). Production of hemolymphopoietic cytokines (IL-6, IL-8, colony-stimulating factors) by normal human astrocytes in response to IL-1 beta and tumor necrosis factor-alpha. J Immunol.

[20] Ehrlich LC, Hu S, Sheng WS, Sutton RL, Rockswold GL, Peterson PK (1998). Cytokine regulation of human microglial cell IL-8 production. J Immunol.

[21] Zheng SG, Wang J, Horwitz DA (2008). Cutting edge: Foxp3^+^CD4^+^CD25^+^ regulatory T cells induced by IL-2 and TGF-β are resistant to Th17 conversion by IL-6. J Immunol.

[22] Ozturk T, Kollhoff A, Anderson AM, Howell JC, Loring DW, Waldrop-Valverde D (2019). Linked CSF reduction of phosphorylated tau and IL-8 in HIV associated neurocognitive disorder. Sci Rep.

[23] Mishra V, Re DB, Le Verche V, Alvarez MJ, Vasciaveo A, Jacquier A (2020). Systematic elucidation of neuron-astrocyte interaction in models of amyotrophic lateral sclerosis using multi-modal integrated bioinformatics workflow. Nat Commun.

[24] Gomez-Pinedo U, Villar-Quiles RN, Galan L, Matias-Guiu JA, Benito-Martin MS, Guerrero-Sola A (2016). Immununochemical markers of the amyloid cascade in the hippocampus in motor neuron diseases. Front Neurol.

[25] Hu WT, Howell JC, Ozturk T, Gangishetti U, Kollhoff AL, Hatcher-Martin JM (2019). CSF cytokines in aging, multiple sclerosis, and dementia. Front Immunol.

[26] Hu WT, Ozturk T, Kollhoff A, Wharton W, Howell JC. Alzheimer's disease neuroimaging I. Higher CSF sTNFR1-related proteins associate with better prognosis in very early Alzheimer's disease. Nat Commun. 2021;12(1):4001. 10.1038/s41467-021-24220-7 PMC8238986 34183654

[27] Howell JC, Watts KD, Parker MW, Wu J, Kollhoff A, Wingo TS (2017). Race modifies the relationship between cognition and Alzheimer's disease cerebrospinal fluid biomarkers. Alzheimers Res Ther.

[28] Benameur K, Agarwal A, Auld SC, Butters MP, Webster AS, Ozturk T (2020). Encephalopathy and encephalitis associated with cerebrospinal fluid cytokine alterations and coronavirus disease, Atlanta, Georgia, USA, 2020. Emerg Infect Dis.

[29] Pane M, Coratti G, Sansone VA, Messina S, Catteruccia M, Bruno C (2021). Type I SMA new natural history: long-term data in nusinersen-treated patients. Ann Clin Transl Neurol.

[30] Rosengren LE, Karlsson JE, Karlsson JO, Persson LI, Wikkelso C (1996). Patients with amyotrophic lateral sclerosis and other neurodegenerative diseases have increased levels of neurofilament protein in CSF. J Neurochem.

[31] Semra YK, Seidi OA, Sharief MK (2002). Heightened intrathecal release of axonal cytoskeletal proteins in multiple sclerosis is associated with progressive disease and clinical disability. J Neuroimmunol.

[32] Gangishetti U, Howell JC, Perrin RJ, Louneva N, Watts KD, Kollhoff A (2018). Non-beta-amyloid/tau cerebrospinal fluid markers inform staging and progression in Alzheimer's disease. Alzheimers Res Ther.

[33] Kester MI, Scheffer PG, Koel-Simmelink MJ, Twaalfhoven H, Verwey NA, Veerhuis R (2012). Serial CSF sampling in Alzheimer's disease: specific versus non-specific markers. Neurobiol Aging.

[34] Huang F, Zhu Y, Hsiao-Nakamoto J, Tang X, Dugas JC, Moscovitch-Lopatin M (2020). Longitudinal biomarkers in amyotrophic lateral sclerosis. Ann Clin Transl Neurol.

[35] Mitchell RM, Freeman WM, Randazzo WT, Stephens HE, Beard JL, Simmons Z (2009). A CSF biomarker panel for identification of patients with amyotrophic lateral sclerosis. Neurology.

[36] Tateishi T, Yamasaki R, Tanaka M, Matsushita T, Kikuchi H, Isobe N (2010). CSF chemokine alterations related to the clinical course of amyotrophic lateral sclerosis. J Neuroimmunol.

[37] Baron P, Bussini S, Cardin V, Corbo M, Conti G, Galimberti D (2005). Production of monocyte chemoattractant protein-1 in amyotrophic lateral sclerosis. Muscle Nerve.

[38] Kuhle J, Lindberg RLP, Regeniter A, Mehling M, Steck AJ, Kappos L (2009). Increased levels of inflammatory chemokines in amyotrophic lateral sclerosis. Eur J Neurol.

[39] Gille B, De Schaepdryver M, Goossens J, Dedeene L, De Vocht J, Oldoni E (2019). Serum neurofilament light chain levels as a marker of upper motor neuron degeneration in patients with amyotrophic lateral sclerosis. Neuropathol Appl Neurobiol.

[40] Huang F, Zhu Y, Hsiao-Nakamoto J, Tang X, Dugas JC, Moscovitch-Lopatin M (2020). Longitudinal biomarkers in amyotrophic lateral sclerosis. Ann Clin Trans Neurol.

[41] Stanga S, Brambilla L, Tasiaux B, Dang AH, Ivanoiu A, Octave JN (2018). A role for GDNF and soluble APP as biomarkers of amyotrophic lateral sclerosis pathophysiology. Front Neurol.

[42] Dirren E, Aebischer J, Rochat C, Towne C, Schneider BL, Aebischer P (2015). SOD1 silencing in motoneurons or glia rescues neuromuscular function in ALS mice. Ann Clin Transl Neurol.

[43] Rindt H, Feng Z, Mazzasette C, Glascock JJ, Valdivia D, Pyles N (2015). Astrocytes influence the severity of spinal muscular atrophy. Hum Mol Genet.

[44] Gogliotti RG, Quinlan KA, Barlow CB, Heier CR, Heckman CJ, Didonato CJ (2012). Motor neuron rescue in spinal muscular atrophy mice demonstrates that sensory-motor defects are a consequence, not a cause, of motor neuron dysfunction. J Neurosci.

[45] Paez-Colasante X, Seaberg B, Martinez TL, Kong L, Sumner CJ, Rimer M (2013). Improvement of neuromuscular synaptic phenotypes without enhanced survival and motor function in severe spinal muscular atrophy mice selectively rescued in motor neurons. PLoS One.

[46] Izumi R, Hino M, Wada A, Nagaoka A, Kawamura T, Mori T (2021). Detailed postmortem profiling of inflammatory mediators expression revealed post-inflammatory alternation in the superior temporal gyrus of schizophrenia. Front Psychiatry.

[47] Horuk R, Martin AW, Wang Z, Schweitzer L, Gerassimides A, Guo H (1997). Expression of chemokine receptors by subsets of neurons in the central nervous system. J Immunol.

[48] La Cognata V, Golini E, Iemmolo R, Balletta S, Morello G, De Rosa C (2021). CXCR2 increases in ALS cortical neurons and its inhibition prevents motor neuron degeneration in vitro and improves neuromuscular function in SOD1G93A mice. Neurobiol Dis.

[49] Whalen MJ, Carlos TM, Kochanek PM, Wisniewski SR, Bell MJ, Clark RS (2000). Interleukin-8 is increased in cerebrospinal fluid of children with severe head injury. Crit Care Med.

[50] Kwon BK, Stammers AM, Belanger LM, Bernardo A, Chan D, Bishop CM (2010). Cerebrospinal fluid inflammatory cytokines and biomarkers of injury severity in acute human spinal cord injury. J Neurotrauma.

[51] Guasp M, Munoz-Sanchez G, Martinez-Hernandez E, Santana D, Carbayo A, Naranjo L (2022). CSF biomarkers in COVID-19 associated encephalopathy and encephalitis predict long-term outcome. Front Immunol.

[52] Kaneko K, Sato DK, Nakashima I, Ogawa R, Akaishi T, Takai Y (2018). CSF cytokine profile in MOG-IgG+ neurological disease is similar to AQP4-IgG+ NMOSD but distinct from MS: a cross-sectional study and potential therapeutic implications. J Neurol Neurosurg Psychiatry.

[53] Choi SS, Lee HJ, Lim I, Satoh J, Kim SU (2014). Human astrocytes: Secretome profiles of cytokines and chemokines. PLoS One.

[54] Jangi M, Fleet C, Cullen P, Gupta SV, Mekhoubad S, Chiao E (2017). SMN deficiency in severe models of spinal muscular atrophy causes widespread intron retention and DNA damage. Proc Natl Acad Sci U S A.

[55] Porterfield V, Khan SS, Foff EP, Koseoglu MM, Blanco IK, Jayaraman S (2020). A three-dimensional dementia model reveals spontaneous cell cycle re-entry and a senescence-associated secretory phenotype. Neurobiol Aging.

[56] Kossmann T, Stahel PF, Lenzlinger PM, Redl H, Dubs RW, Trentz O (1997). Interleukin-8 released into the cerebrospinal fluid after brain injury is associated with blood-brain barrier dysfunction and nerve growth factor production. J Cereb Blood Flow Metab.

