# Supplementary material for: CSF IL-8 Associated with Response to Gene Therapy in a Case Series of Spinal Muscular Atrophy
Source: Neurotherapeutics. 2022 Oct 26;20(1):245–53. doi: 10.1007/s13311-022-01305-9 (PMC9607706; doi:10.1007/s13311-022-01305-9)
Supplement: Supplementary file 1 — Supplementary file1 (DOCX 18 KB) [file 13311_2022_1305_MOESM1_ESM.docx]

**Supplementary Material**

CSF IL-8 associated with response to gene therapy in a case series of Spinal Muscular Atrophy

Sumit Verma, M.D., Kelsey Perry, Raj Razdan, MS, J. Christina Howell, M.A., Alice L. Dawson, M.A., William T. Hu, M.D., Ph.D.

**Supplementary Table 1.** Demographic and clinical factors associated with longitudinal outcomes in CHOP INTEND, CMAP_APB_, CMAP_ADM_, and weight. Sex was not a significant factor in any of the outcomes.

| **Outcome: CHOP INTEND** | **B (95% CI)** | **P** |
| --- | --- | --- |
| Younger age | 3.798 (-0.734, 8.329) | 0.099 |
| Time (months) | -0.178 (-0.754, 0.398) | 0.541 |
| **Baseline CHOP INTEND** | **0.697 (0.525, 0.870)** | **<0.001** |
| **Younger age X Time** | **1.198 (0.462, 1.934)** | **0.002** |

| **Outcome: CMAP_APB_** | **B (95% CI)** | **P** |
| --- | --- | --- |
| Younger age | 0.074 (-0.155, 0.302) | 0.522 |
| Time (months) | 0.003 (-0.035, 0.041) | 0.889 |
| **Baseline CMAP_APB_** | **0.928 (0.741, 1.115)** | **<0.001** |
| **Younger age X Time** | **0.071 (0.023, 0.119)** | **0.004** |

| **Outcome: CMAP_ADM_** | **B (95% CI)** | **P** |
| --- | --- | --- |
| Younger age | -0.018 (-0.236, 0.200) | 0.867 |
| Time (months) | 0.004 (-0.019, 0.028) | 0.702 |
| **Baseline CMAP_ADM_** | **0.831 (0.518, 1.144)** | **<0.001** |
| Younger age X Time | 0.031 (-0.001, 0.063) | 0.060 |
|  |  |  |
| **Outcome: Weight** | **B (95% CI)** | **P** |
| Time (months) | 0.248 (0.146, 0.350) | <0.001 |
| Baseline weight | 0.915 (0.802, 1.027) | <0.001 |

**Supplementary Table 2.** Longitudinal CSF proteins associated with SMA clinical outcomes. For each clinical outcome, age group, sex, and time were entered as independent variables (time as fixed and random). Time-dependent analyte levels and – when appropriate – analyte X age group were also entered as fixed variables, with the latter term to adjust for age-dependent relationship between CSF proteins and clinical outcomes. False Discovery Rate of 5% was used to identify proteins associated with prognosis before and/or after correction for age group (≤24 vs. >24 months).

|  | **CHOP INTEND** | **CMAP_APB_** | **CMAP_ADM_** | **Weight** |
| --- | --- | --- | --- | --- |
| **NfL**  **NfL X Age group** | F=0.695, p=0.411  **F=9.578, p=0.004** | F=0.757, p=0.391 | F=1.178, p=0.287 | F=1.970, p=0.176 |
| **IL-8**  **IL-8 X Age group** | **F=17.503, p<0.001**  F=5.222, p=0.027 | F=3.670, p=0.064 | F=1.412, p=0.244 | F=0.001, p=0.981 |
| **IP-10** | F=1.758, p=0.199 | F=3.012, p=0.092 | F=9.064, p=0.008 | F=0.001, p=0.980 |
| **Fractalkine** | F=0.902, p=0.350 | F=0.013, p=0.909 | F=0.073, p=0.789 | F=1.963, p=0.175 |
| **MCP1**  **MCP X Age group** | **F=6.902, p=0.012**  F=3.183, p=0.082 | F=1.605, p=0.214 | F=1.151, p=0.295 | F=0.138, p=0.716 |
| **sAPPα**  **sAPPα X Age group** | **F=5.194, p=0.028** | F=0.328, p=0.570 | F=0.730, p=0.400 | F=0.916, p=0.345  F=5.410, p=0.025 |
| **sAPPβ** | F=0.024, p=0.877 | F=0.583, p=0.451 | F=0.797, p=0.384 | F=0.122, p=0.729 |
